# Association between plant-based diets and metabolic health status in adolescents with overweight and obesity

**DOI:** 10.1038/s41598-022-17969-4

**Published:** 2022-08-12

**Authors:** Elahe Mokhtari, Saeideh Mirzaei, Ali Asadi, Masoumeh Akhlaghi, Parvane Saneei

**Affiliations:** 1 Department of Community Nutrition, School of Nutrition and Food Science, Nutrition and Food Security Research Center, Students’ Research Committee, Isfahan University of Medical Sciences, Isfahan, Iran; 2 Department of Community Nutrition, School of Nutrition and Food Science, Shiraz University of Medical Sciences, Shiraz, Iran; 3 Department of Exercise Physiology, School of Physical Education and Sport Sciences, University of Tehran, Tehran, Iran; 4 Department of Community Nutrition, School of Nutrition and Food Sciences, Shiraz University of Medical Sciences, Shiraz, Iran; 5 Department of Community Nutrition, School of Nutrition and Food Science, Nutrition and Food Security Research Center, Isfahan University of Medical Sciences, PO Box 81745-151, Isfahan, Iran

**Keywords:** Medical research, Risk factors

## Abstract

The association of plant-based diets with health status is underestimated in pediatrics. We aimed to examine the relation between plant-based diets (including overall plant-based index (PDI), healthy plant-based (hPDI) and unhealthy plant-based (uPDI)) and metabolic health status in Iranian adolescents with overweight/obesity. We conducted a cross-sectional study on 203 adolescents with overweight/obesity (12–18 years old) selected by a multistage cluster random-sampling method. Usual dietary intakes were assessed through a validated 147-item food frequency questionnaire (FFQ). Anthropometric indices and blood pressure values were measured and fasting blood samples were drawn. For classification of participants into metabolically healthy obese (MHO) or metabolically unhealthy obese (MUO) groups, two methods of International Diabetes Federation (IDF) and combination of IDF with Homeostasis Model Assessment Insulin Resistance (HOMA-IR) were applied. No significant association was observed between higher adherence to PDI and odds of MUO status defined by both IDF and IDF/HOMA-IR strategies. After adjustments for all potential confounders, adolescents in the highest tertile of hPDI, compared with those in the lowest tertile, had 85% (95% CI 0.05–0.43) and 84% (95% CI 0.05, 0.52) lower odds of being MUO based on IDF and IDF/HOMA-IR criteria, respectively. Greater adherence to uPDI was associated with odd of 3.95 (95% CI 1.41, 11.12) and 4.06 (95% CI 1.31, 12.57) of being MUO based on IDF and IDF/HOMA-IR definitions, after considering all potential confounders. Stratified analysis revealed that these associations were stronger in girls and overweight subjects. Adherence to healthy plant-based foods was inversely associated with odds of MUO status in Iranian adolescents. In contrast, unhealthy plant-based diets was directly associated with MUO in pediatrics. Further studies with prospective nature, are required to affirm these results.

## Introduction

Prevalence of childhood obesity has drastically increased and this disorder has become one of the most important public health problems worldwide^1,2^. In 2015, a total number of 107.7 million children were known to be obese, resulted in a prevalence of 23% of childhood obesity and overweight in the globe^3,4^. Childhood obesity is not only epidemic in developed countries (such as European countries and USA), but also it is prevalent in developing countries^5^. In Iran, it is estimated that almost 4 million children and adolescents will have excess body weight by 2025^6^. Childhood obesity could cause many health problems, such as hypertension, type 2 diabetes mellitus (T2D), coronary heart diseases (CHD), high cholesterol, stroke, cancer, asthma, sleep disorders and liver disease^7,8^. Nevertheless, not all children and adolescents with excess body weight display these complications. A distinct subgroup of children with obesity called “metabolically healthy obese” (MHO) are less prone to develop metabolic disturbances and display a “favorable” metabolic profile, while other children with obesity defined as “metabolically unhealthy overweight or obese” (MUO), are more likely to develop metabolic complications^9,10^. The health status of many MHO children might shift to MUO in adulthood^11^ So, the affecting factors such as genetic and lifestyle risk factors, including dietary intake and physical activity, can possibly differentiate obesity phenotypes^12^. Preventing this transition might be a key intervention target to maintain MHO status later in life. Recently, nutritional epidemiology has focused on examining the impact of dietary patterns such as the plant-based diets on health outcomes (including, T2D and CHD)^13,14^, instead of evaluating the effect of nutrients or individual food groups.

Findings from previous investigations on intake of food groups or dietary patterns in relation to metabolic health status and cardio-metabolic implications are contradictory^15–20^. In a cross-sectional study on overweight Latino youth, investigators had demonstrated that consumption of specific types of vegetables was associated with positive metabolic outcomes including reduced risk of visceral and liver fat and type 2 diabetes^15^; intake of non-starchy vegetables was associated with lower liver fat deposition and dark green or bright orange/yellow vegetables intake resulted in lower visceral fat and improved insulin sensitivity^15^. Another cross-sectional study showed that association between sugar intake and adiposity or metabolic risk might depend on the source of the sugar. Indeed, sugar taken from fruit could inversely be associated with the adiposity index; whereas, beverage sugar had an adverse association with metabolic risk in youth^16^. Boon et al. examined the association between snacking patterns and body mass index (BMI) among the adolescents. They found that more snack intake (including milk, soft drinks and caffeinated beverages), was associated with more energy and carbohydrate intake, as compared to protein or fat intake; however, higher carbohydrate intake through different types of consumed snacks was not significantly associated with BMI in school-age adolescents^17^. In contrast, results from some other investigations showed significant unfavorable association between sugar-sweetened beverage intake and cardiometabolic health outcomes including elevated serum triglyceride, fasting blood glucose, insulin, insulin resistance and low HDL-c levels^18^ or higher waist circumference (WC) and BMI^19^ among young adolescents. Moreover, a dietary pattern that was high in energy density, high in fat and low in fiber could result in adiposity in childhood and adolescence^20^. Although previous studies provided information about the association between plant-based diets and some cardiometabolic risk factors^15,16^, to the best of our knowledge, there was no study that examined the relation between plant-based diets and metabolic health status in children and adolescents. So, the current study was conducted to evaluate the association of plant-based diets with metabolic health status in Iranian adolescents.

## Methods

### Participants

This cross-sectional study was conducted on a representative sample of adolescents living in Iran in 2020. The sample size of the current study was calculated based on previous published investigations^21,22^, that showed approximately 60% of adolescents with overweight and obesity in Iran suffer from MUO. Thus, with a power 80%, type I error of 0.05, desired confidence interval of 0.95, and precision (d) of 7%, the minimum required sample size was estimated to be 188 individuals. A stratified, multi-stage cluster sampling design was used to randomly select participants from 5 different districts of the city of Isfahan, Iran. Sixteen schools were randomly selected and BMI was calculated for all students of these schools; then, students with overweight or obesity (based on age-sex-specific BMI percentiles^23^) were invited to participate in the current investigation. Adolescents with different socioeconomic status were considered in the sample by using this method. Individuals with the following criteria (based on their self-reports) were not included in the current analysis: (1) those who had genetic or endocrine disorders (such as type 1 diabetes mellitus, hypothyroidism or Cushing’s syndrome), (2) those who were on a weight-loss diet, (3) those who were taking vitamin and mineral supplementation and medications which might effect on body weight, blood glucose, lipid profile or hypertension. Prior to enrollment, detailed information on the research purposes and procedures has been given to eligible students and their parents. To avoid stigmatization of adolescents with overweight or obesity, the aim of the study was described as evaluating metabolic health status of individuals without referring to body weight or using the terms of “overweight”, “obesity”, or “fat mass”. So, we recruited 203 adolescents with overweight/obesity (102 girls and 101 boys) with the age of 12 to 18 years old in the current study. Written informed consent was obtained from all participants and their parents. The study protocol was approved by the local Ethics Committee of Isfahan University of Medical Sciences (Ethical number: IR.ARI.MUI.REC.1400.071).

### Assessment of dietary intakes

Data of dietary intake of participants were collected by a validated 147-item food frequency questionnaire (FFQ)^24^. Previous investigations showed that this FFQ could accurately indicate dietary intakes and their relations with various diseases in Iranian adolescents^25,26^. Thus, reasonable validity and reliability in order to assess foods and nutrients in Iranian pediatrics were documented for this tool. A trained nutritionist has completed FFQs and requested the participants to report their frequency of consumption (based on daily, weekly, or monthly) and amount of consumption (based on standard common portion size) of food items in the preceding year. Then, portion sizes of consumed foods were converted to grams/day, using household measures^27^. The grams of food intakes were entered into Nutritionist IV software to examine nutrient intake data. The applied Nutritionist IV software was based on USDA food composition database; contents of some Iranian foods were also added to it.

### Plant-based dietary indices

Using dietary data, we created 3 different indices: an overall plant-based diet index (PDI), a healthful plant-based diet index (hPDI (, and an unhealthful plant-based diet index (uPDI). We used the procedure previously described by Satija et al.^13,14,28^, to create these indices. First, we created 18 food groups (by summing up the grams of consumed food items) based on the nutrient and culinary similarities within larger categories of healthy and less healthy plant foods and animal foods. Healthy plant food groups were consisted of whole grains, vegetables, fruits, nuts, legumes, vegetable oils, and tea or coffee, while less healthy plant food groups included fruit juices, refined grains, potatoes, sugar-sweetened beverages, and sweets or desserts. In addition, animal food groups included animal fats, dairy, eggs, fish or seafood, different types of meat and miscellaneous animal-based foods. As fatty acid composition of margarine has changed over time from high trans-fat to high unsaturated fat, we excluded this item from the indices. Moreover, trans-fatty acids were not included in the indices because it was not possible to determine the intact amount of it in foods. However, we controlled for margarine and hydrogenated vegetable oil intake—as the main source of trans-fatty acid—in multivariable analysis. Each of the 18 food groups (as gram/day) was divided into quintiles of consumption and quintiles were positively or negatively scored. With positive scores, participants with highest consumption (quintile 5) of the food group were received a score of 5, while subjects with lowest intake (quintile 1) were received a score of 1. With reverse scores, individuals in the highest quintile of the food group were given a score of 1, whereas participants in the lowest quintile got a score of 5. To create PDI, all plant food groups were given positive scores and animal food groups were given reverse scores. For creating hPDI, healthy plant foods were given positive scores, whereas less-healthy plant food groups and animal food groups were given negative scores. To create uPDI, less-healthy plant food groups were given positive scores, while healthy plant food groups and animal food groups were given reverse scores. For each participant, Scores of these 18 food groups were summed up to obtain the indices, with a theoretical range of 18 (lowest possible score) to 90 (highest possible score).

### Assessment of anthropometric indices and cardio-metabolic risk factors

All measurements were done by trained nutritionists. Weight was measured in minimal clothing and without shoes using a calibrated electronic scale (Seca Instruments, Germany) to the nearest 0.1 kg. Standing height was also measured without shoes using a stadiometer (to the nearest 0.1 cm). BMI was calculated as weight (kg) divided by height squared (m^2^). Then, students were classified as normal, adolescents with overweight or obesity based on the age-sex-specific BMI percentiles defined by World Health Organization (WHO) for adolescents^23^. Waist circumference (WC) was recorded twice by using an unstretched flexible anthropometric tape (to the nearest 0.1 cm), midway between the lowest rib and the superior border of the iliac crest, after a normal expiration and without any pressure on the body surface. Then, the mean value of two measured values for each student was considered as WC. For measurement of blood pressure, after a rest period of 15 min systolic blood pressure (SBP) and diastolic blood pressure (DBP) were measured twice, at the right arm, by using a mercury sphygmomanometer with a suitable cuff size. The average of the two measurements for each participant was considered as SBP and DBP. Blood samples were drawn from all participants in a sitting position, according to the standard protocol after 12 h overnight fasting. The blood samples were collected in vacuum tubes and centrifuged within 30–45 min after collection. Fasting blood glucose (FBG) concentration was measured with an enzymatic colorimetric method by the use of glucose oxidase (Pars Azmoon commercial kits, Tehran, Iran). Serum insulin was measured using ELISA kits (Diagnostic Biochem Canada Inc.). Homeostasis Model Assessment Insulin Resistance (HOMA-IR) was additionally calculated to estimate insulin resistance (IR) through the use of the following formula: HOMA-IR = [ (fasting insulin (mU/L) × FBG (mmol/L)]/22.5. Serum HDL-c concentration was measured with phosphotungstic acid, after precipitation of the apolipoprotein B-containing lipoproteins (Pars Azmoon commercial kits, Tehran, Iran). Serum triglyceride concentration was also assayed using triacylglycerol kits by enzymatic colorimetric tests with glycerol phosphate oxidase (Pars Azmoon commercial kits, Tehran, Iran).

### Assessment of metabolic status

Two methods were applied for the classification of participants into MHO or MUO. The first method was based on the modified International Diabetes Federation (IDF) criteria^29^. Based on IDF definition, participants who had two or more of the following risk factors were classified as MUO subjects: increased triglycerides (TG) (≥ 150 mg/dL), decreased HDL-c (< 40 mg/dL for the age of < 16 y, and < 50 mg/dL in females/ < 40 mg/dL in males for the age of ≥ 16 y), increased fasting blood glucose (≥ 100 mg/dL) and increased blood pressure (≥ 130/85 mmHg). In this definition, those with one/no defined risk factors were considered as MHO adolescents. In the second method, insulin resistance defined as HOMA-IR score, was added to the IDF criteria items that were used in the first classification^30^. Thus, students with two or more mentioned metabolic risk factors and HOMA-IR score ≥ 3.16 were deemed to MUO individuals and those with HOMA-IR < 3.16 were considered as MHO adolescents. The cut-off value of 3.16 was selected for HOMA-IR, according to some previous studies on children and adolescents obesity^31–33^. It is worth noting that WC was not included in these two definitions for MHO/MUO.

### Assessment of other variables

To evaluate physical activity level of participants, Physical Activity Questionnaire for Adolescents (PAQ-A) questionnaire was used which contains nine items on various activities assessing physical activity of the last week^34^. Items 1 to 8 of this questionnaire are about the usual activity of adolescents and the ninth is about unusual activity of adolescents during the previous week. Then, scores were summed up and adolescents were categorized into active (score ≥ 3), low active (3 < score ≤ 2), sedentary (or not having an orderly week activity) (score < 2), on the basis of their total scores. Moreover, to evaluate socioeconomic status (SES) of students, a validated demographic questionnaire was used by trained investigators^35^ based on the following variables: parental job, family size, parental education level, having cars in the family, having computers/laptops, having personal room and taking trips in the year. In addition, age, gender, history of diseases and use of medications and dietary supplements of participants were recorded through a demographic questionnaire.

### Statistical analysis

The Kolmogorov–Smirnov test was applied to examine the normality of quantitative variables. The continuous variables were presented as mean ± SD/SE and qualitative variables as frequency (percentage). All participants were categorized into tertiles of PDI, hPDI and uPDI, based on the scores of these patterns. To compare quantitative and categorical variables between tertiles of PDI, hPDI and uPDI, one-way analysis of variance (ANOVA) and χ^2^ test was respectively used. Age-, sex- and energy-adjusted dietary intakes of participants across tertiles PDI, hPDI and uPDI of were evaluated by analysis of covariance (ANCOVA). To identify the association between PDI, hPDI and uPDI and MUO status, multivariable logistic regression was applied. The odds ratio (OR) and 95% confidence interval (CI) for MUO status were calculated in crude and adjusted models. In the first model, adjustments were done for sex and age and energy intake. In the second model, further adjustment for physical activity levels, and socioeconomic status was made. In the third model, intake of margarine and hydrogenated vegetable oil were added to adjustments. In the last model, further adjustment for BMI was made. In all models, the first tertile of PDI, hPDI or uPDI was considered as the reference category. The overall trend of OR across increasing PDI, hPDI and uPDI tertiles was examined by considering tertiles of each dietary pattern as a continuous variable. SPSS software version 19 (IBM, Chicago, IL) was used for all analyses. P-values < 0.05 (two-sided) were considered as statistically significant.

### Ethical approval and consent to participate

The study procedure was performed according to declaration of Helsinki and STROBE checklist. All participants provided informed written consent. The study protocol was approved by the local Ethics Committee of Isfahan University of Medical Sciences.

## Results

General characteristics and cardiometabolic factors of study participants across tertiles of PDI, hPDI and uPDI are presented in Table 1. There was no significant difference in general characteristics and cardiometabolic variables among tertiles of PDI. In comparison to those in the lowest tertiles of hPDI, adolescents in the highest tertile had lower weight, height, WC, systolic and diastolic blood pressure, FBG, insulin, HOMA-IR index, TG, and higher HDL-c (P < 0.05). In addition, those in the top category of hPDI were more likely to be physically active (P < 0.05). There were no significant differences in sex, age, BMI and socioeconomic status among tertiles of hPDI. Adolescents in the highest tertile of the uPDI were more likely to be girls, be less physically active, have low socioeconomic status, lower HDL-c and have higher FBG and TG compared with those in the lowest tertile (P < 0.05). There were no significant differences in other characteristics of participants among tertiles of uPDI.

**Table 1:** General characteristics and cardiometabolic factors of study participants across tertiles of PDI, hPDI and uPDI.

	Tertiles of PDI				Tertiles of hPDI				Tertiles of uPDI			
	T1 (n = 61)	T2 (n = 71)	T3 (n = 71)	P-value^1^	T1 (n = 72)	T2 (n = 65)	T3 (n = 66)	P-value^1^	T1 (n = 62)	T2 (n = 74)	T3 (n = 67)	P-value^1^
Range	< 51	51–55	> 55	–	< 50	50–57	> 57	–	< 49	49–58	> 58	–
**Sex, n (%)**												
Boys	28 (45.9)	35 (49.3)	38 (53.5)	0.68	42 (58.3)	29 (44.6)	30 (45.5)	0.19	40 (64.5)	37 (50.0)	24 (35.8)	0.01
Girls	33 (54.1)	36 (50.7)	33 (46.5)		30 (41.7)	36 (55.4)	36 (54.5)		22 (35.5)	37 (50.0)	43 (64.2)	
Age (year)	14.1 ± 1.62	13.9 ± 1.69	13.9 ± 1.53	0.67	13.9 ± 1.50	14.1 ± 1.62	14.0 ± 1.73	0.75	13.9 ± 1.82	14.0 ± 1.54	14.0 ± 1.49	0.91
Weight (kg)	72.4 ± 9.89	72.1 ± 11.06	75.8 ± 13.19	0.12	76.4 ± 10.96	72.2 ± 11.31	71.6 ± 12.11	0.03	72.4 ± 12.17	72.9 ± 10.96	75.1 ± 11.75	0.35
Height (cm)	164.3 ± 7.97	162.7 ± 7.64	164.0 ± 8.26	0.49	166.0 ± 8.28	162.4 ± 8.00	162.3 ± 6.99	0.01	163.6 ± 7.95	163.9 ± 8.09	163.3 ± 7.89	0.91
BMI^3^ (kg/m^2^)	26.8 ± 2.61	27.1 ± 2.81	28.1 ± 3.96	0.05	27.6 ± 2.66	27.3 ± 2.85	27.1 ± 4.09	0.64	27.0 ± 3.32	27.0 ± 3.15	28.1 ± 3.19	0.09
Waist circumference (cm)	89.1 ± 7.09	90.1 ± 7.07	91.6 ± 9.26	0.20	92.6 ± 6.91	89.0 ± 7.17	89.2 ± 9.17	0.01	89.4 ± 9.59	90.4 ± 6.72	91.2 ± 7.51	0.46
**Physical activity levels, n (%)**												
Sedentary	23 (37.7)	28 (39.4)	38 (53.5)	0.07	41 (56.9)	36 (55.4)	12 (18.2)	< 0.001	12 (19.4)	28 (37.8)	49 (73.1)	< 0.001
Low-activity	25 (41.0)	25 (35.2)	27 (38.0)		28 (38.9)	23 (35.4)	26 (39.4)		24 (38.7)	36 (48.6)	17 (25.4)	
Active	13 (21.3)	18 (25.4)	6 (8.5)		3 (4.2)	6 (9.2)	28 (42.4)		26 (41.9)	10 (13.5)	1 (1.5)	
**Socioeconomic status**^**2**^**, n (%)**												
Low	21 (34.4)	17 (23.9)	21 (29.6)	0.25	23 (31.9)	23 (35.4)	13 (19.7)	0.33	12 (19.4)	17 (23.0)	30 (44.8)	0.01
Medium	30 (49.2)	32 (45.1)	28 (39.4)		31 (43.1)	25 (38.5)	34 (51.5)		27 (43.5)	36 (48.6)	27 (40.3)	
High	10 (16.4)	22 (31.0)	22 (31.0)		18 (25.0)	17 (26.2)	19 (28.8)		23 (37.1)	21 (28.4)	10 (14.9)	
Systolic blood pressure (mmHg)	110.7 ± 16.94	113.2 ± 10.09	113.9 ± 24.87	0.58	116.6 ± 10.85	113.7 ± 17.86	107.5 ± 23.63	0.01	112.8 ± 10.59	112.4 ± 21.18	113.0 ± 20.75	0.98
Diastolic blood pressure (mmHg)	73.3 ± 10.53	73.4 ± 6.52	73.7 ± 15.36	0.98	75.8 ± 5.90	75.0 ± 6.33	69.5 ± 17.33	0.01	73.1 ± 6.80	72.4 ± 12.52	75.1 ± 13.27	0.36
Fasting blood glucose level (mg/dL)	97.2 ± 9.09	98.5 ± 9.29	98.6 ± 7.12	0.58	101.9 ± 9.53	98.2 ± 6.97	94.0 ± 6.67	< 0.001	94.6 ± 7.87	98.5 ± 6.70	101.0 ± 9.70	< 0.001
Insulin (μUI/mL)	19.7 ± 15.54	19.7 ± 10.89	21.7 ± 11.58	0.56	24.0 ± 14.28	19.8 ± 9.09	17.2 ± 13.00	0.01	18.5 ± 13.41	20.4 ± 13.72	22.3 ± 10.45	0.25
HOMA-IR index	4.79 ± 3.86	4.87 ± 2.92	5.37 ± 3.09	0.54	6.02 ± 3.53	4.88 ± 2.55	4.07 ± 3.37	0.01	4.46 ± 3.64	4.99 ± 3.29	5.58 ± 2.84	0.15
Triglycerides (mg/dL)	118.9 ± 65.23	122.3 ± 69.51	124.3 ± 65.50	0.90	140.2 ± 74.02	127.6 ± 65.06	96.5 ± 50.53	< 0.001	106.2 ± 59.01	117.4 ± 68.06	141.7 ± 67.55	0.01
HDL cholesterol (mg/dL)	45.9 ± 7.83	45.2 ± 7.48	43.6 ± 8.37	0.23	43.5 ± 7.70	43.7 ± 7.59	47.4 ± 7.99	0.01	47.5 ± 8.34	45.1 ± 6.62	42.1 ± 8.08	0.01

Values are Mean ± SD; unless indicated. Abbreviations: BMI: Body Mass Index; HOMA-IR: Homeostasis Model Assessment Insulin Resistance; HDL-c: high-density lipoprotein cholesterol.

^1^P-value for one-way ANOVA test and χ2 test for quantitative and categorical variables, respectively.

^2^Socioeconomic status (SES) score was evaluated based on parental education level, parental job, family size, having car in the family, having computer/laptop, having personal room and having travel by using demographic questionnaire.

Dietary intakes of study participants across tertiles of PDI, hPDI and uPDI are shown in Table 2. Adolescents in the highest tertile of PDI, compared to those in the lowest tertile, had higher intake of energy, carbohydrate, vitamin C, vitamin E and total dietary fiber and lower intake of protein. Among tertiles of hPDI, those in the highest tertile in comparison with the lowest tertile had lower intake of energy and higher intake of vitamin C and total dietary fiber. There were no significant differences in carbohydrate, protein, fat and vitamin E intake among tertiles of hPDI. Compared with adolescents in the lowest tertile of uPDI, those in the highest tertile had lower intake of protein, fat, vitamin C and total dietary fiber and higher intake of carbohydrate. No significant differences were seen in energy and vitamin E intake among tertiles of uPDI.

**Table 2:** Dietary intakes (energy and macro/micro nutrients) of study participants across tertiles of PDI, hPDI and uPDI.

	Tertiles of PDI				Tertiles of hPDI				Tertiles of uPDI			
	T1 (n = 61)	T2 (n = 71)	T3 (n = 71)	P-value^1^	T1 (n = 72)	T2 (n = 65)	T3 (n = 66)	P-value^1^	T1 (n = 62)	T2 (n = 74)	T3 (n = 67)	P-value^1^
Range	< 51	51–55	> 55	–	< 50	50–57	> 57	–	< 49	49–58	> 58	–
Energy, kcal	2746.2 ± 68.45	2871.8 ± 63.37	3011.9 ± 63.40	0.02	3083.7 ± 62.03	2783.5 ± 65.06	2762.1 ± 64.53	< 0.001	2907 ± 70.13	2852.1 ± 63.25	2895.0 ± 67.46	0.82
Protein, % of E	15.2 ± 0.25	14.4 ± 0.23	13.5 ± 0.23	< 0.001	14.0 ± 0.24	14.2 ± 0.25	14.8 ± 0.25	0.05	15.5 ± 0.24	14.3 ± 0.21	13.2 ± 0.23	< 0.001
Carbohydrate, % of E	56.8 ± 0.66	57.9 ± 0.60	59.9 ± 0.61	0.01	58.3 ± 0.63	58.3 ± 0.65	58.2 ± 0.65	0.99	55.7 ± 0.63	58.7 ± 0.57	60.3 ± 0.61	< 0.001
Fat, % of E	29.4 ± 0.67	29.0 ± 0.62	28.2 ± 0.62	0.42	28.9 ± 0.63	29.0 ± 0.65	28.7 ± 0.65	0.94	30.5 ± 0.66	28.5 ± 0.59	27.6 ± 0.63	0.01
Cholesterol, mg	327.4 ± 11.86	288.4 ± 10.87	236.8 ± 10.99	< 0.001	283.4 ± 11.98	281.8 ± 12.31	280.9 ± 12.25	0.99	333.9 ± 11.67	281.2 ± 10.53	235.0 ± 11.23	< 0.001
SFA, gr	30.1 ± 0.71	26.9 ± 0.65	25.4 ± 0.66	< 0.001	28.2 ± 0.70	27.6 ± 0.72	26.2 ± 0.72	0.12	29.1 ± 0.73	27.4 ± 0.66	25.7 ± 0.70	0.01
MUFA, gr	28.9 ± 0.88	27.8 ± 0.81	26.1 ± 0.81	0.06	27.3 ± 0.84	27.5 ± 0.86	28.0 ± 0.85	0.85	31.3 ± 0.82	26.7 ± 0.74	25.0 ± 0.79	< 0.001
PUFA, gr	26.5 ± 1.02	28.8 ± 0.94	29.9 ± 0.95	0.05	27.7 ± 0.97	28.9 ± 1.00	29.0 ± 0.99	0.61	28.6 ± 1.03	28.3 ± 0.93	28.6 ± 0.99	0.97
Vitamin C, mg	120.7 ± 7.50	129.1 ± 6.87	149.2 ± 6.95	0.02	98.6 ± 6.27	135.4 ± 6.44	170.1 ± 6.41	< 0.001	173.2 ± 6.55	134.0 ± 5.91	96.6 ± 6.30	< 0.001
Vitamin A, RAE	1072.0 ± 81.66	1052.0 ± 74.86	1193.3 ± 75.66	0.37	814.4 ± 71.67	1146.8 ± 73.69	1388.3 ± 73.30	< 0.001	1468.4 ± 73.25	1124.5 ± 66.08	754.6 ± 0.45	< 0.001
Thiamin, mg	2.57 ± 0.04	2.69 ± 0.04	2.66 ± 0.04	0.09	2.64 ± 0.04	2.66 ± 0.04	2.64 ± 0.04	0.88	2.55 ± 0.04	2.65 ± 0.04	2.73 ± 0.04	0.01
Riboflavin, mg	2.52 ± 0.07	2.32 ± 0.06	2.10 ± 0.06	< 0.001	2.19 ± 0.07	2.26 ± 0.07	2.46 ± 0.07	0.02	2.73 ± 0.06	2.26 ± 0.06	1.95 ± 0.06	< 0.001
Niacin, mg	27.5 ± 0.45	27.7 ± 0.41	27.6 ± 0.42	0.96	28.1 ± 0.42	27.9 ± 0.43	26.6 ± 0.43	0.03	26.0 ± 0.42	27.5 ± 0.38	29.1 ± 0.40	< 0.001
Vitamin B6, mg	1.60 ± 0.05	1.61 ± 0.05	1.65 ± 0.05	0.83	1.50 ± 0.05	1.63 ± 0.05	1.75 ± 0.05	0.01	1.86 ± 0.05	1.65 ± 0.04	1.37 ± 0.05	< 0.001
Vitamin E, mg	27.1 ± 1.47	31.3 ± 1.35	32.2 ± 1.36	0.03	29.7 ± 1.40	30.8 ± 1.44	30.7 ± 1.44	0.84	28.7 ± 1.48	30.6 ± 1.34	31.6 ± 1.43	0.37
Folate, mcg	309.2 ± 13.06	314.9 ± 11.97	324.7 ± 12.10	0.68	253.8 ± 10.53	319.1 ± 10.83	382.7 ± 10.77	< 0.001	409.2 ± 9.68	310.7 ± 8.73	237.5 ± 9.31	< 0.001
Vitamin B12, mcg	5.14 ± 0.18	4.49 ± 0.17	3.78 ± 0.17	< 0.001	4.27 ± 0.18	4.46 ± 0.19	4.59 ± 0.19	0.46	5.59 ± 0.16	4.34 ± 0.14	3.47 ± 0.15	< 0.001
Magnesium, mg	292.6 ± 8.17	283.9 ± 7.49	288.7 ± 7.57	0.74	258.9 ± 7.10	289.4 ± 7.30	318.9 ± 7.26	< 0.001	342.1 ± 6.26	287.3 ± 5.65	239.3 ± 6.02	< 0.001
Zinc, mg	11.4 ± 0.29	10.6 ± 0.26	10.0 ± 0.27	0.01	10.1 ± 0.27	10.8 ± 0.28	11.1 ± 0.28	0.03	12.2 ± 0.25	10.6 ± 0.22	9.2 ± 0.24	< 0.001
Selenium, mcg	0.09 ± 0.00	0.10 ± 0.00	0.10 ± 0.00	0.67	0.10 ± 0.00	0.10 ± 0.00	0.08 ± 0.00	0.01	0.08 ± 0.00	0.10 ± 0.00	0.10 ± 0.00	0.01
Total fiber, gr	18.2 ± 0.61	18.7 ± 0.56	21.3 ± 0.56	< 0.001	15.8 ± 0.47	19.6 ± 0.48	23.3 ± 0.48	< 0.001	23.1 ± 0.51	19.6 ± 0.46	15.9 ± 0.49	< 0.001
Sodium, mg	4189.4 ± 148.17	3979.6 ± 135.83	3825.5 ± 137.29	0.21	4266.1 ± 135.41	4111.0 ± 139.21	3565.8 ± 138.49	0.01	3717.9 ± 146.27	3984.9 ± 131.96	4243.7 ± 140.67	0.04
Potassium, mg	3416.3 ± 116.52	3277.6 ± 106.81	3445.0 ± 107.97	0.50	2916.7 ± 99.30	3388.7 ± 102.09	3870.1 ± 101.55	< 0.001	4161.6 ± 88.69	3359.1 ± 80.01	2673.2 ± 85.29	< 0.001

Values are Mean ± SE. Energy intake was adjusted for age and gender; all other values were adjusted for age, gender and energy intake (by the use of ANCOVA). Abbreviations: E: energy intake; SFA, Saturated fatty acids; MUFA, Monounsaturated fatty acids; PUFA, Polyunsaturated fatty acids.

^1^P-value obtained from ANCOVA test for adjustment of energy intake.

**Figure 1:**
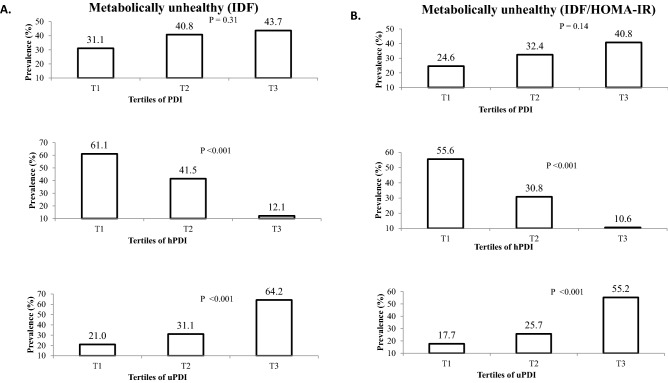
Prevalence of MUO across tertiles of PDI, hPDI and uPDI. (**A**) Based on IDF definition, (**B**) based on IDF/HOMA-IR definition.

The prevalence of MUO adolescents across tertiles of PDI, hPDI and uPDI -based on IDF and IDF/HOMA-IR criteria- are presented in Fig. 1. Based on IDF definition for metabolic health status, the prevalence of MUO in the highest tertile of PDI was not significantly different with the lowest tertile (43.7 vs. 31.1%, P = 0.31). However, participants in the top tertile of hPDI compared with those in the bottom tertile, had lower prevalence of being MUO (12.1 vs. 61.1%, P < 0.001). On the other hand, greater adherence to uPDI was associated with higher prevalence of MUO (third tertile vs. first tertile of uPDI: 64.2 vs. 21.0%, P < 0.001). According to the second definition of metabolic health status based on IDF/HOMA-IR, the same findings were obtained (MUO prevalence in T3 vs. T1 of PDI: 40.8 vs. 24.6%, P = 0.14; T3 vs.T1 of hPDI: 10.6 vs. 55.6%, P < 0.001; T3 vs.T1 of uPDI: 55.2 vs. 17.7%, P < 0.001).

Crude and multivariable adjusted odds ratio and 95% CI for MUO phenotype across tertiles of PDI, hPDI and uPDI are presented in Table 3. According to IDF criteria, no significant association was observed between PDI categories and odds of MUO in adolescents, in crude model (OR_T3 vs. T1_: 1.71; 95% CI 0.84–3.51). Adjustments for potential confounders did not change this non-significant relation (OR_T3 vs. T1_: 1.03; 95% CI 0.43–2.48). Among hPDI tertiles, those in the third tertile had 91% lower odds for MUO, based on IDF criteria in the crude model (OR_T3 vs. T1_: 0.09; 95% CI 0.04–0.21). This association was significant after adjustment for all potential confounders; such that, adolescents in the top category of hPDI compared with the bottom category had 85% lower odds of MUO status in the fully-adjusted model (OR_T3 vs. T1_: 0.15; 95% CI 0.05–0.43). On the other hand, greater adherence to uPDI was associated with increased odds of MUO, based on IDF criteria (OR_T3 vs. T1_: 6.75; 95% CI 3.07–14.87). In the fully-adjusted model, the association remained significant; highest tertile of uPDI was associated with higher odds of MUO profile (OR_T3 vs. T1_: 3.95; 95% CI 1.41–11.12). When we evaluated all these associations with MUO based on IDF/HOMA-IR criteria, the same findings were obtained.

**Table 3:** Multivariate adjusted odds ratio (OR) and 95% confidence interval (CI) for MUO phenotype across tertiles of PDI, hPDI and uPDI.

	Tertiles of PDI				Tertiles of hPDI				Tertiles of uPDI			
	T1 (n = 61)	T2 (n = 71)	T3 (n = 71)	P-trend^1^	T1 (n = 72)	T2 (n = 65)	T3 (n = 66)	P-trend^1^	T1 (n = 62)	T2 (n = 74)	T3 (n = 67)	P-trend^1^
**MUO phenotype based on IDF criteria**												
Cases (n)	19	29	31		44	27	8		13	23	43	
Crude	1 (Ref.)	1.53 (0.74, 3.13)	1.71 (0.84 3.51)	0.15	1 (Ref.)	0.45 (0.23, 0.90)	0.09 (0.04, 0.21)	< 0.001	1 (Ref.)	1.70 (0.78, 3.73)	6.75 (3.07, 14.87)	< 0.001
Model 1	1 (Ref.)	1.33 (0.63, 2.80)	1.32 (0.62, 2.83)	0.49	1 (Ref.)	0.55 (0.26, 1.14)	0.10 (0.04, 0.25)	< 0.001	1 (Ref.)	1.95 (0.84, 4.53)	8.55 (3.56, 20.50)	< 0.001
Model 2	1 (Ref.)	1.63 (0.69, 3.84)	1.08 (0.46, 2.55)	0.92	1 (Ref.)	0.53 (0.24, 1.19)	0.18 (0.07, 0.48)	0.01	1 (Ref.)	1.31 (0.51, 3.35)	3.96 (1.43, 10.98)	0.01
Model 3	1 (Ref.)	1.60 (0.68, 3.80)	1.07 (0.45, 2.57)	0.92	1 (Ref.)	0.55 (0.24, 1.24)	0.18 (0.07, 0.49)	0.01	1 (Ref.)	1.29 (0.50, 3.36)	4.00 (1.43, 11.19)	0.01
Model 4	1 (Ref.)	1.61 (0.68, 3.82)	1.03 (0.43, 2.48)	0.99	1 (Ref.)	0.53 (0.23, 1.22)	0.15 (0.05, 0.43)	< 0.001	1 (Ref.)	1.33 (0.51, 3.48)	3.95 (1.41, 11.12)	0.01
**MUO phenotype based on HOMA-IR criteria**												
Cases (n)	15	23	29		40	20 (30.8)	7		11	19	37	
Crude	1 (Ref.)	1.47 (0.68, 3.16)	2.12 (1.00, 4.49)	0.05	1 (Ref.)	0.36 (0.18, 0.72)	0.10 (0.04, 0.24)	< 0.001	1 (Ref.)	1.60 (0.70, 3.69)	5.72 (2.54, 12.86)	< 0.001
Model 1	1 (Ref.)	1.20 (0.54, 2.67)	1.51 (0.68, 3.37)	0.30	1 (Ref.)	0.49 (0.23, 1.03)	0.12 (0.05, 0.31)	< 0.001	1 (Ref.)	2.00 (0.80, 5.02)	8.41 (3.12, 21.31)	< 0.001
Model 2	1 (Ref.)	1.37 (0.56, 3.35)	1.22 (0.49, 3.00)	0.70	1 (Ref.)	0.45 (0.20, 1.02)	0.22 (0.07, 0.62)	0.01	1 (Ref.)	1.39 (0.50, 3.89)	4.04 (1.35, 12.04)	0.01
Model 3	1 (Ref.)	1.33 (0.53, 3.30)	1.20 (0.48, 2.99)	0.73	1 (Ref.)	0.44 (0.18, 1.03)	0.21 (0.07, 0.63)	0.01	1 (Ref.)	1.35 (0.48, 3.86)	4.10 (1.35, 12.46)	0.01
Model 4	1 (Ref.)	1.35 (0.54, 3.39)	1.13 (0.45, 2.85)	0.84	1 (Ref.)	0.42 (0.18, 1.01)	0.16 (0.05, 0.52)	0.01	1 (Ref.)	1.44 (0.49, 4.22)	4.06 (1.31, 12.57)	0.01

All values are odds ratios and 95% confidence intervals. Model 1: Adjusted for age, sex, and energy intake. Model 2: Additionally, adjusted for physical activity and socioeconomic status (evaluated based on parental education level, parental job, family size, having car in the family, having computer/laptop, having personal room and having travel by using demographic questionnaire). Model 3: Additionally, adjusted for margarine and trans fatty acids. Model 4: Additionally, adjusted for Body Mass Index (BMI).

^1^Obtained by the use of tertiles of PDI, hPDI, or uPDI as an ordinal variable in the model.

Crude and multivariate adjusted odds ratio (OR) and 95% confidence interval (CI) for MUO phenotype across tertiles of PDI, hPDI and uPDI, stratified by gender are shown in Table 4. Stratified analysis showed no significant association between PDI and MUO based on both metabolic health status definitions, either in boys or in girls. Higher adherence to hPDI in girls was associated with decreased odds of MUO profile based on IDF definition; such that, after adjustment for all potential confounders, a 94% lower odds was observed for those in the last tertile vs. the first one (95% CI 0.01–0.31). Although in the crude model a significant lower odds of being MUO (based on IDF criteria) was seen in the top category of hPDI (OR_T3 vs. T1_: 0.15; 95% CI 0.05–0.47), this association disappeared in the fully-adjusted model (OR_T3 vs. T1_: 0.27; 95% CI 0.05–1.46) among boys. In both genders, higher adherence to uPDI was associated with greater odds of being MUO based on IDF criteria either in the crude or adjusted models (fully-adjusted model for girls: OR_T3 vs. T1_: 8.17; 95% CI 1.24–53.63; for boys: OR_T3 vs. T1_: 7.00; 95% CI 1.32–37.13). When HOMA-IR was added to IDF criteria, an inverse association between hPDI and MUO was still observed in girls (in fully-adjusted model: OR_T3 vs. T1_: 0.10; 95% CI 0.01–0.68). However, after adjustment for all potential confounders, significant association between hPDI and MUO disappeared in boys. Higher adherence to uPDI was significantly associated with increased odds of MUO phenotype based on IDF/HOMA-IR definition either in boys or in girls, both in crude and adjusted model for age and energy intake. However, after adjustment for all potential confounders, only a marginally significant relation was observed in boys (OR_T3 vs. T1_: 5.26; 95% CI 1.00–27.67).

**Table 4:** Multivariate adjusted odds ratio (OR) and 95% confidence interval (CI) for MUO phenotype across tertiles of PDI, hPDI and uPDI, stratified by gender.

	Tertiles of PDI				Tertiles of hPDI				Tertiles of uPDI			
	T1	T2	T3	P-trend^1^	T1	T2	T3	P-trend^1^	T1	T2	T3	P-trend^1^
**MUO phenotype based on IDF criteria**												
Girls (participants/cases)	33/12	33/15	36/15		30/20	36/19	36/3		22/4	37/12	43/26	
Crude	1 (Ref.)	1.25 (0.47, 3.30)	1.46 (0.54, 3.91)	0.45	1 (Ref.)	0.56 (0.21, 1.52)	0.05 (0.01, 0.19)	< 0.001	1 (Ref.)	2.16 (0.60, 7.80)	6.88 (1.98, 23.88)	0.01
Model 1	1 (Ref.)	0.94 (0.33, 2.67)	0.90 (0.30, 2.67)	0.84	1 (Ref.)	0.58 (0.19, 1.76)	0.04 (0.01, 0.17)	< 0.001	1 (Ref.)	3.20 (0.77, 13.30)	13.00 (2.95, 57.33)	< 0.001
Model 2	1 (Ref.)	0.79 (0.23, 2.70)	0.66 (0.19, 2.32)	0.52	1 (Ref.)	0.50 (0.14, 1.73)	0.06 (0.01, 0.31)	0.01	1 (Ref.)	1.53 (0.28, 8.26)	6.49 (1.10, 38.09)	0.01
Model 3	1 (Ref.)	0.80 (0.23, 2.73)	0.68 (0.19, 2.46)	0.56	1 (Ref.)	0.50 (0.14, 1.77)	0.06 (0.01, 0.31)	0.01	1 (Ref.)	1.53 (0.28, 8.45)	6.57 (1.11, 38.84)	0.01
Model 4	1 (Ref.)	0.79 (0.23, 2.72)	0.69 (0.19, 2.48)	0.57	1 (Ref.)	0.50 (0.14, 1.76)	0.06 (0.01, 0.31)	0.01	1 (Ref.)	1.74 (0.30, 10.06)	8.17 (1.24, 53.63)	0.01
Boys (participants/cases)	28/7	35/14	38/16		42/24	29/8	30/5		40/9	37/11	24/17	
Crude	1 (Ref.)	2.00 (0.67, 5.95)	2.18 (0.75, 6.37)	0.17	1 (Ref.)	0.29 (0.10, 0.79)	0.15 (0.05, 0.47)	0.01	1 (Ref.)	1.46 (0.52, 4.06)	8.37 (2.65, 26.45)	< 0.001
Model 1	1 (Ref.)	1.89 (0.61, 5.89)	1.85 (0.60, 5.73)	0.33	1 (Ref.)	0.36 (0.12, 1.05)	0.18 (0.06, 0.60)	0.01	1 (Ref.)	1.59 (0.54, 4.69)	9.68 (2.88, 32.59)	< 0.001
Model 2	1 (Ref.)	3.15 (0.83, 12.02)	1.64 (0.45, 5.96)	0.57	1 (Ref.)	0.47 (0.14, 1.61)	0.34 (0.09, 1.35)	0.11	1 (Ref.)	1.34 (0.41, 4.46)	4.20 (1.03, 17.10)	0.05
Model 3	1 (Ref.)	2.77 (0.69, 11.15)	1.81 (0.47, 6.98)	0.44	1 (Ref.)	0.46 (0.12, 1.69)	0.41 (0.09, 1.85)	0.21	1 (Ref.)	2.25 (0.58, 8.69)	5.16 (1.09, 24.43)	0.04
Model 4	1 (Ref.)	2.59 (0.63, 10.53)	1.58 (0.40, 6.24)	0.59	1 (Ref.)	0.43 (0.12, 1.58)	0.27 (0.05, 1.46)	0.10	1 (Ref.)	3.03 (0.71, 12.99)	7.00 (1.32, 37.13)	0.02
**MUO phenotype based on HOMA-IR criteria**												
Girls (participants/cases)	33/9	36/9	33/14		30/17	36/12	36/3		22/2	37/9	43/21	
Crude	1 (Ref.)	0.89 (0.30, 2.61)	1.97 (0.70, 5.51)	0.19	1 (Ref.)	0.38 (0.14, 1.04)	0.07 (0.02, 0.28)	< 0.001	1 (Ref.)	3.21 (0.63, 16.51)	9.55 (1.98, 45.96)	0.01
Model 1	1 (Ref.)	0.64 (0.20, 2.06)	1.19 (0.37, 3.77)	0.72	1 (Ref.)	0.43 (0.14, 1.30)	0.07 (0.02, 0.32)	< 0.001	1 (Ref.)	4.96 (0.81, 30.40)	16.65 (2.73, 101.4)	0.01
Model 2	1 (Ref.)	0.45 (0.11, 1.75)	0.90 (0.23, 3.52)	0.96	1 (Ref.)	0.26 (0.07, 0.95)	0.11 (0.02, 0.76)	0.01	1 (Ref.)	2.25 (0.25, 19.94)	8.86 (0.98, 80.29)	0.02
Model 3	1 (Ref.)	0.43 (0.11, 1.70)	0.88 (0.22, 3.56)	0.94	1 (Ref.)	0.25 (0.07, 0.95)	0.11 (0.02, 0.74)	0.01	1 (Ref.)	1.99 (0.22, 17.80)	8.33 (0.92, 75.27)	0.02
Model 4	1 (Ref.)	0.45 (0.11, 1.81)	0.87 (0.22, 3.53)	0.91	1 (Ref.)	0.24 (0.06, 0.92)	0.10 (0.01, 0.68)	0.01	1 (Ref.)	1.94 (0.21, 17.82)	7.95 (0.81, 77.95)	0.03
Boys (participants/cases)	28/6	35/14	38/15		42/23	29/8	30/4		40/9	37/10	24/16	
Crude	1 (Ref.)	2.44 (0.79, 7.55)	2.39 (0.79, 7.28)	0.15	1 (Ref.)	0.32 (0.11, 0.87)	0.13 (0.04, 0.43)	< 0.001	1 (Ref.)	1.28 (0.45, 3.60)	6.89 (2.23, 21.27)	0.01
Model 1	1 (Ref.)	2.37 (0.73, 7.74)	2.04 (0.62, 6.66)	0.30	1 (Ref.)	0.43 (0.14, 1.26)	0.17 (0.05, 0.58)	0.01	1 (Ref.)	1.38 (0.46, 4.14)	7.93 (2.39, 26.31)	0.01
Model 2	1 (Ref.)	4.19 (1.04, 16.93)	1.92 (0.50, 7.40)	0.46	1 (Ref.)	0.57 (0.17, 2.00)	0.28 (0.07, 1.16)	0.08	1 (Ref.)	1.14 (0.33, 3.89)	3.34 (0.82, 13.57)	0.10
Model 3	1 (Ref.)	3.84 (0.89, 16.51)	2.14 (0.52, 8.76)	0.35	1 (Ref.)	0.58 (0.15, 2.16)	0.32 (0.07, 1.58)	0.15	1 (Ref.)	1.87 (0.47, 7.53)	4.11 (0.86, 19.72)	0.08
Model 4	1 (Ref.)	3.66 (0.84, 15.90)	1.91 (0.46, 8.00)	0.46	1 (Ref.)	0.54 (0.14, 2.04)	0.20 (0.03, 1.21)	0.07	1 (Ref.)	2.44 (0.55, 10.83)	5.26 (1.00, 27.67)	0.05

All values are odds ratios and 95% confidence intervals. Model 1: Adjusted for age and energy intake. Model 2: Additionally, adjusted for physical activity and socioeconomic status (evaluated based on parental education level, parental job, family size, having car in the family, having computer/laptop, having personal room and having travel by using demographic questionnaire). Model 3: Additionally, adjusted for margarine and trans fatty acids. Model 4: Additionally, adjusted for Body Mass Index (BMI).

^1^Obtained by the use of tertiles of PDI, hPDI, or uPDI as an ordinal variable in the model.

Crude and multivariate adjusted odds ratio (OR) and 95% confidence interval (CI) for MUO phenotype across tertiles of PDI, hPDI and uPDI, stratified by BMI categories are shown in Table 5. Our analyses revealed that either adolescents with overweight or obesity in higher tertile of PDI were more likely to be MUO based on both metabolic health status definitions, but this association was not statistically significant. Compared with the lowest adherence, highest adherence to hPDI was associated with a decreased likelihood of being MUO (based on IDF criteria), in crude model either in individuals with overweight (OR_T3 vs. T1_: 0.02; 95% CI 0.01–0.13) or obesity (OR_T3 vs. T1_: 0.25; 95% CI 0.08–0.75). But, after adjustment for all potential confounders, only a significant relation was observed in overweight individuals (OR_T3 vs. T1_: 0.02; 95% CI 0.01–0.23). Among tertiles of uPDI, in crude model, higher uPDI tertile was associated with increased odds of 10.46 and 4.09 for MUO phenotype based on IDF criteria in adolescents with overweight (95% CI 2.94–37.19) and obesity (95% CI 1.38–12.09), respectively. After adjustment for all potential confounders, these significant associations remained significant in both BMI categories (for subjects with overweight: OR_T3 vs. T1_: 8.42; 95% CI 1.09–64.85; for subjects with obesity: OR_T3 vs. T1_: 5.33; 95% CI 1.22–23.28). According to IDF/HOMA-IR criteria for MUO, in crude model and after adjustment for age, sex and energy intake, higher adherence to hPDI was associated with lower odds of MUO both in adolescents with overweight and those with obesity. However, in fully-adjusted model, the relation was significant only in overweight adolescents (OR_T3 vs. T1_: 0.08; 95% CI 0.01–0.86). For uPDI, higher adherence was associated with higher odds of MUO defined by IDF/HOMA-IR criteria in both individuals with overweight and those with obesity, in crude model (for adolescents with overweight: OR_T3 vs. T1_: 12.71; 95% CI 2.55–63.20; for adolescents with obesity: OR_T3 vs. T1_: 3.21; 95% CI 1.11–9.29). After adjustment for all potential confounders, these relations were diminished and no longer significant.

**Table 5:** Multivariate adjusted odds ratio (OR) and 95% confidence interval (CI) for MUO phenotype across tertiles of PDI, hPDI and uPDI, stratified by BMI categories.

	Tertiles of PDI				Tertiles of hPDI				Tertiles of uPDI			
	T1	T2	T3	P-trend^1^	T1	T2	T3	P-trend^1^	T1	T2	T3	P-trend^1^
**MUO phenotype based on IDF criteria**												
Overweight												
(Participants/cases)	35/7	37/11	32/10		24/16	37/10	43/2		38/4	37/8	29/16	
Crude	1 (Ref.)	1.69 (0.57, 5.02)	1.82 (0.60, 5.55)	0.30	1 (Ref.)	0.19 (0.06, 0.57)	0.02 (0.01, 0.13)	< 0.001	1 (Ref.)	2.35 (0.64, 8.59)	10.46 (2.94, 37.19)	< 0.001
Model 1	1 (Ref.)	1.56 (0.50, 4.83)	1.54 (0.45, 5.26)	0.49	1 (Ref.)	0.19 (0.05, 0.67)	0.02 (0.003, 0.11)	< 0.001	1 (Ref.)	3.18 (0.77, 13.22)	14.72 (3.46, 62.55)	< 0.001
Model 2	1 (Ref.)	1.15 (0.28, 4.72)	0.98 (0.23, 4.24)	0.97	1 (Ref.)	0.20 (0.05, 0.81)	0.04 (0.01, 0.26)	0.01	1 (Ref.)	1.11 (0.19, 6.41)	4.68 (0.78, 28.09)	0.04
Model 3	1 (Ref.)	0.88 (0.20, 3.89)	0.74 (0.15, 3.66)	0.71	1 (Ref.)	0.18 (0.04, 0.80)	0.02 (0.01, 0.23)	0.01	1 (Ref.)	1.86 (0.26, 13.44)	8.42 (1.09, 64.85)	0.02
Obese												
(Participants/cases)	26/12	34/18	39/21		48/28	28/17	23/6		24/9	37/15	38/27	
Crude	1 (Ref.)	1.31 (0.47, 3.65)	1.36 (0.50, 3.68)	0.56	1 (Ref.)	1.10 (0.43, 2.86)	0.25 (0.08, 0.75)	0.03	1 (Ref.)	1.14 (0.40, 3.27)	4.09 (1.38, 12.09)	0.01
Model 1	1 (Ref.)	1.14 (0.39, 3.32)	1.13 (0.40, 3.20)	0.84	1 (Ref.)	1.36 (0.49, 3.76)	0.30 (0.10, 0.93)	0.07	1 (Ref.)	1.25 (0.40, 3.91)	5.86 (1.71, 20.10)	0.01
Model 2	1 (Ref.)	1.89 (0.55, 6.43)	1.34 (0.42, 4.25)	0.68	1 (Ref.)	1.29 (0.43, 3.81)	0.43 (0.12, 1.51)	0.30	1 (Ref.)	1.50 (0.42, 5.35)	4.55 (1.08, 19.23)	0.03
Model 3	1 (Ref.)	1.69 (0.49, 5.87)	1.37 (0.43, 4.40)	0.64	1 (Ref.)	1.23 (0.40, 3.80)	0.46 (0.13, 1.63)	0.32	1 (Ref.)	1.67 (0.46, 6.12)	5.33 (1.22, 23.28)	0.02
**MUO phenotype based on HOMA-IR criteria**												
Overweight												
(Participants/cases)	35/4	37/7	32/9		24/13	37/5	43/2		38/2	37/6	29/12	
Crude	1 (Ref.)	1.81 (0.48, 6.82)	3.03 (0.83, 11.08)	0.09	1 (Ref.)	0.13 (0.04, 0.46)	0.04 (0.01, 0.21)	< 0.001	1 (Ref.)	3.48 (0.66, 18.52)	12.71 (2.55, 63.20)	0.01
Model 1	1 (Ref.)	1.73 (0.44, 6.79)	2.49 (0.61, 10.18)	0.21	1 (Ref.)	0.17 (0.05, 0.62)	0.05 (0.01, 0.26)	< 0.001	1 (Ref.)	6.26 (0.97, 40.18)	20.06 (3.28, 122,79)	< 0.001
Model 2	1 (Ref.)	1.20 (0.23, 6.28)	1.51 (0.26, 8.62)	0.64	1 (Ref.)	0.16 (0.04 (0.70)	0.14 (0.02, 1.05)	0.02	1 (Ref.)	1.65 (0.19, 14.28)	4.36 (0.53, 35.74)	0.10
Model 3	1 (Ref.)	1.01 (0.18, 5.55)	1.20 (0.17, 6.98)	0.92	1 (Ref.)	0.12 (0.02, 0.60)	0.08 (0.01, 0.86)	0.01	1 (Ref.)	2.28 (0.21, 25.42)	6.88 (0.64, 73.84)	0.06
Obese												
(Participants/cases)	26/11	34/16	39/20		48/27	28/15	23/5		24/9	37/13	38/25	
Crude	1 (Ref.)	1.21 (0.43, 3.39)	1.44 (0.53, 3.90)	0.48	1 (Ref.)	0.90 (0.35, 2.29)	0.22 (0.07, 0.68)	0.01	1 (Ref.)	0.90 (0.31, 2.62)	3.21 (1.11, 9.29)	0.02
Model 1	1 (Ref.)	1.00 (0.34, 2.95)	1.16 (0.40, 3.33)	0.77	1 (Ref.)	1.15 (0.42, 3.13)	0.26 (0.08, 0.84)	0.04	1 (Ref.)	0.98 (0.30, 3.16)	4.71 (1.38, 16.08)	0.01
Model 2	1 (Ref.)	1.53 (0.45, 5.15)	1.33 (0.42, 4.22)	0.66	1 (Ref.)	1.08 (0.37, 3.17)	0.32 (0.09, 1.20)	0.15	1 (Ref.)	1.22 (0.33, 4.48)	3.89 (0.92, 16.54)	0.05
Model 3	1 (Ref.)	1.40 (0.41, 4.81)	1.39 (0.43, 4.46)	0.60	1 (Ref.)	1.09 (0.36, 3.38)	0.35 (0.09, 1.32)	0.18	1 (Ref.)	1.29 (0.34, 4.85)	4.31 (0.99, 18.68)	0.04

All values are odds ratios and 95% confidence intervals. Model 1: Adjusted for age, sex, and energy intake. Model 2: Additionally, adjusted for physical activity and socioeconomic status (evaluated based on parental education level, parental job, family size, having car in the family, having computer/laptop, having personal room and having travel by using demographic questionnaire). Model 3: Additionally, adjusted for margarine and trans fatty acids.

^1^Obtained by the use of tertiles of PDI, hPDI, or uPDI as an ordinal variable in the model.

## Discussion

In this cross-sectional study, three indices of plant-based diet, healthy plant-based diet and unhealthy plant-based diet were studied among Iranian adolescents with overweight/obesity. Our results indicated that healthy plant based diet was associated with lower odds of MUO status. On the other hand, odds of being MUO increased by higher adherence to unhealthy plant-based diet. These associations were independent of the criteria used for definition of metabolic health status. Moreover, findings from stratified analysis revealed that greater adherence to hPDI was associated with lower odds for MUO and higher uPDI adherence was related to higher odds of MUO, more considerably among girls and overweight adolescents in comparison with boys and adolescents with obesity. To our knowledge, this was the first investigation that evaluated the association between plant-based diets with metabolic status in adolescents with overweight or obesity.

Childhood obesity is an increasing concern with respect to the health and well-being of pediatrics^1,2^. Hence, effective strategies are needed to prevent and control obesity and its complications in children^9–11^. Dietary intakes are among the most effective factors of obesity status. So, educating and encouraging adolescents to improve their diet quality by increasing the intake of healthy plant-based foods and limiting the intake of unhealthy plant foods could be helpful in order to delay the onset of obesity-related metabolic complications.

Several prior studies have examined the association between fruit and vegetable consumption and different metabolic risks in children and adolescents^36–39^. Kepper et al. have reported that children who have greater access to fast foods and lower access to fruits and vegetables might experience a higher risk for developing obesity^37^. A 4-week randomized trial documented benefits of plant-based diets, including decreased overweight, obesity and cardiovascular risk, in both children and their parents^38^. Moreover, Van Hulst et al. showed that low consumption of saturated fats and greater intake of vegetable and fruit were associated with better insulin sensitivity in children as they enter into puberty and these healthy dietary choices may help at-risk children to prevent later development of type 2 diabetes^39^. Field et al. have studied a large group of American preadolescents and adolescents in a prospective cohort investigation and reported that although the recommendation for consumption of at least five servings of fruits and vegetables might be associated with reduced risk of some chronic diseases such as coronary heart diseases and cancer, it would be not based on a beneficial impact on BMI regulation^36^. Some other investigations evaluated the association between some particular plant-based foods and different metabolic risks. Cook et al. in a cross-sectional study confirmed that consumption of non-starchy vegetables was associated with lower liver fat deposition, and improvement of insulin sensitivity would be resulted by consuming dark green or bright orange or yellow vegetables^15^. Although none of previous studies investigated the association between plant based diets (categorized as healthy and unhealthy plants) and metabolic risk factors among children or adolescents, some relationships were studied in adults. Satija et al. recommended that hPDI was inversely associated with lower risk of T2D incidence and in contrast, less healthy plant based diets had positive association with incidence of the disease^13^. Furthermore, another cohort study revealed that greater adherence to a healthy plant-based diet was associated with lower risk of coronary heart diseases (CHD) and those who consumed more unhealthy plant foods might experience higher risk of CHD^14^. Contradictory findings of earlier investigations might be due to different types of study design, study population and method of data collection or the outcome of interests among investigations.

Several probable mechanisms may elucidate the observed desirable or adverse associations. Higher adherence to healthy plant-based foods would lead to a specific diet with high dietary fiber, antioxidants, micronutrient content including magnesium and unsaturated fat, and low saturated fat, heme iron content and energy intake. Such a diet could have so many beneficial effects on metabolic health such as helping weight loss/maintenance, glycemic control and insulin regulation, as well as improving lipid profile, decreasing blood pressure and inflammation, and developing more favorable diet-gut microbiome interactions^13,14,28^. Furthermore, several prospective studies have documented that dietary fiber could be inversely associated with levels of inflammatory markers^40,41^. Moreover, energy intake might be affected by dietary fiber in particular through changes in hunger and satiety cues. Absorption of water by soluble fibers could lead to higher amount of chewing, viscous gel formation and slower gut transit time and higher satiety^42^. Previous animal and epidemiologic studies have revealed that antioxidants such as polyphenols might have beneficial effects on glucose metabolism through decreasing oxidative stress and improving endothelial function^43^. As mentioned above, another less well understood mechanism of healthy plant-based diets could be through the gut microbiome. Gut microbial environment could get boosted and as a result facilitates the metabolism of fiber and polyphenols and improves the metabolism of bile acids, choline and l-carnitine, and amino acids^44^. On the other hand, consuming more unhealthy plant based foods would lead to a diet with high glycemic load and index, added sugar, calorie content and low dietary fiber, micronutrients, unsaturated fats, and antioxidants which could adversely affect the above-mentioned pathways^13,14,28^. Higher glycemic load and index of such an unhealthy diet might cause a decline in satiety and increase in hunger signals^45,46^. Additionally, unhealthy plant-based diets would also have a high level of added sugar, which has been shown to have a strong association with higher risk of weight gain and T2D^44,47^. Finally, higher content of added sugar and refined carbohydrates in this diet could lead to higher post-prandial insulin increment, which is known to be involved in progression of MHO to MUO^48^.

Current study has some strength and weaknesses. First, the novelty of the study was evaluation of three plant-based diets with metabolic health status in a sample of Iranian adolescents from different socioeconomic status. Second, two different criteria for defining metabolic health status were applied. Third, several potential confounders were considered in the analysis. However, some limitations should be noted in the interpretation of our results. We could not establish causality, because of the cross-sectional design of our study. Therefore, confirming the causal associations between plant-based diets and metabolic health status should be noticed in future prospective studies. Furthermore, dietary assessment was performed by the use of an FFQ which might cause misclassification of participants in terms of adherence to dietary patterns, although the applied FFQ was previously validated^24–26^. Data collection was also carried out in an interview setting for dietary intakes, which might be associated with social desirability bias. Recall bias and other potential reporting bias might additionally have influenced the findings. Furthermore, despite controlling for several confounders, the possible effects of residual confounders (such as dietary habits, puberty and sleep health) should also be taken into account. Finally, although we measured BMI and WC to define obesity and abdominal obesity, we could not measure body composition and fat distribution which would be involved in metabolic health status.

In conclusion, our results demonstrated that more adherence to healthy plant-based diets was inversely associated with odds of being MUO in Iranian adolescents, while unhealthy plant-based diets was related to an increased likelihood of MUO status. Therefore, higher adherence to healthy plant foods may improve metabolic health status of adolescents. Further studies, in particular with prospective nature, are required to confirm these findings.

## Abbreviations

PDI: Plant-based diet index
hPDI: Healthful plant-based diet index
uPDI: Unhealthful plant-based diet index
FFQ: Food frequency questionnaire
OR: Odds ratios
95% CI: 95% Confidence interval
BMI: Body mass index
MHO: Metabolically healthy obese
MUO: Metabolically unhealthy obese
IDF: International Diabetes Federation
HOMA-IR: Homeostasis model assessment insulin resistance
IR: Insulin resistance
PAQ-A: Physical activity questionnaire for adolescents
SES: Socioeconomic status
SFA: Saturated fatty acids
WHO: World Health Organization
WC: Waist circumference
BP: Blood pressure
SBP: Systolic blood pressure
DBP: Diastolic blood pressure
TG: Triglycerides
HDL-c: High density lipoprotein cholesterol
FBG: Fasting blood glucose
ANCOVA: Analysis of covariance
SPSS: Statistical package for the social sciences
SD: Standard deviation
SE: Standard error
T2D: Type 2 diabetes mellitus
CHD: Coronary heart diseases
